# Prevalence of autoimmune diseases in patients with type 1 diabetes: a scoping review

**DOI:** 10.31744/einstein_journal/2025RW1222

**Published:** 2025-04-17

**Authors:** Carlos Antonio Negrato, Rebecca Zerbinatti Pereira, Livia Domingos de Moraes Pimentel Porto, Ylana Walleska Santos Santana, Aline Kimmy Ikemoto Sato, Vitor Casoto de Melo, Miguel Luz Vilela Engel Vieira, Marilia de Brito Gomes

**Affiliations:** 1 Faculdade de Medicina de Bauru Universidade de São Paulo Bauru SP Brazil Faculdade de Medicina de Bauru, Universidade de São Paulo, Bauru, SP, Brazil.; 2 Universidade do Estado do Rio de Janeiro Rio de Janeiro RJ Brazil Universidade do Estado do Rio de Janeiro, Rio de Janeiro, RJ, Brazil.

**Keywords:** Diabetes mellitus, type 1, Autoimmune diseases, Thyroiditis, autoimmune, Celiac disease, Addison disease

## Abstract

**Objective:**

To evaluate the prevalence of autoimmune diseases in patients with type 1 diabetes mellitus.

**Methods:**

This scoping review was conducted following the Joanna Briggs Institute guidelines and the Preferred Reporting Items for Systematic Reviews and Meta-Analyses Extension for Scoping Reviews (PRISMA-ScR) tool to ensure methodological rigor. We systematically searched PubMed, Embase, Scopus, Lilacs, and Web of Science databases to identify relevant literature published between 2018 and 2023.

**Results:**

Twenty-four studies were included, mostly single-center studies from six continents, with varying study designs: 16 cross-sectional, seven retrospective, and one prospective cohort. The most prevalent autoimmune diseases found among patients with type 1 diabetes mellitus enrolled in these studies were autoimmune thyroiditis (5.5-41.2%), celiac disease (0.45-24.8%), rheumatoid arthritis (0.4-5.1%), and primary adrenal insufficiency (0.6-2.6%).

**Conclusion:**

Autoimmune thyroiditis and celiac disease were the most prevalent autoimmune diseases in patients with type 1 diabetes mellitus. As the complexity of managing type 1 diabetes mellitus increases in the presence of multiple autoimmune comorbidities, further studies are required to elucidate the relationship between type 1 diabetes mellitus and different autoimmune pathologies. A deeper understanding of these associations will guide the development of public health policies, screening strategies, and educational initiatives tailored to the specific needs of this population.

## INTRODUCTION

### Rationale

Type 1 *diabetes mellitus* (T1DM) results from inadequate insulin production by the endocrine pancreas.^1^ Type 1 *diabetes mellitus* can be classified based on its etiopathogenesis into immune-mediated T1DM (type 1A or T1DMA) and idiopathic T1DM (type 1B or T1DMB). In T1DMA, the immune system targets and destroys insulin-producing pancreatic beta cells, driven by specific serum autoantibodies, including anti-insulin (IAA), anti-glutamic acid decarboxylase (anti-GAD65), anti-islet cells (ICA512), anti-zinc transporter 8 (ZnT8), and anti-tyrosine phosphatases IA-2 and IA-2B. In contrast, T1DMB is characterized by the absence of these autoantibodies.^2,3^

Similar to other autoimmune diseases, T1DM exhibits a genetic predisposition in its pathogenesis. Several loci are linked to an increased risk of developing T1DM, particularly the human leukocyte antigen (HLA) region on chromosome 6p21. This region plays a critical role in the immune system by recognizing and presenting a wide range of antigens to T cells. The HLA region is highly polymorphic and encodes the structural synthesis of class I and II molecules. Among class II haplotypes, HLA-DR3, HLA-DR4, and HLA-DQ, as well as their combination, are more frequently found in white individuals with T1DM. However, other genes have been identified in different populations, highlighting the polygenic nature of this disease.^4,5^

The mechanisms through which genetic susceptibility affects T1DM development remain to be elucidated. However, several environmental triggers, such as infections, have been proposed to be associated with disease onset.^6^ Molecular mimicry between some viral antigens, the release of pro-inflammatory cytokines triggered by these infections, and the resulting proinflammatory state contribute to the immune system activation observed in T1DM.^7^

Evidence suggests that molecules from the HLA system, when in contact with self-antigens and in the presence of autoantibodies, activate T cell-mediated cellular destruction, leading to direct tissue damage.^6,8^ Other theories suggest that the destruction of beta cells is due to regulatory failure, in which cellular suppression and activation mechanisms become unbalanced, leading to the loss of T cell self-tolerance.^9,10^

In the insulitis process, which is characterized by the destruction of beta cells, macrophages present self-antigens to CD4 T lymphocytes, triggering the autoimmune response observed in T1DM. The activation of these macrophages initiates the secretion of cytokines that promote cell migration and the secretion of free radicals toxic to beta cells. This process is characterized by a predominance of CD8 T lymphocyte activity and induction of cell apoptosis. CD4 T lymphocytes also participate in this process by secreting cytokines that promote the proliferation and differentiation of T and B lymphocytes and macrophages, thereby enhancing autoimmune destruction.^11^

Similarly, individuals with abnormalities in the mechanisms that induce or maintain self-tolerance may mount immune responses against autologous antigens, making them more prone to developing other autoimmune diseases compared to the general population. The most frequently found autoimmune diseases in individuals with T1DM are thyroid autoimmune disease, celiac disease, Addison’s disease, vitiligo, and rheumatoid arthritis. There may be a common genetic susceptibility between these conditions, as observed in the HLA-DQ haplotype, which is present in both T1DM and celiac disease.^4^ Other studies have also highlighted the role of anti-beta cell antibodies as a risk factor for these autoimmune diseases.^12,13^

The combination of T1DM with other autoimmune diseases can make glycemic control more difficult to achieve. Additionally, it may increase the risk of hypoglycemia and elevate long-term cardiovascular risk, owing to the presence of a chronic inflammatory state.^14^ Microvascular complications, such as neuropathy and retinopathy, have also shown a higher prevalence in individuals with T1DM associated with other autoimmune diseases, except for thyroid autoimmune disease.^14^

Considering the higher prevalence of autoimmune diseases in the population with T1DM as well as the increased risk of complications resulting from this coexistence, which is frequently diagnosed late, it is believed that knowing the existence of these associations would lead to greater health surveillance, contributing to the creation of effective protocols for the early detection of these diseases.

## OBJECTIVE

This review aimed to analyze the prevalence of autoimmune diseases in patients with type 1 *diabetes mellitus*.

## METHODS

This scoping review was conducted following the standards of the Joanna Briggs Institute. The Preferred Reporting Items for Systematic Reviews and Meta-Analyses (PRISMA) tool adapted for scoping reviews (PRISMA-ScR) was utilized to ensure methodological rigor. This tool includes a checklist of 20 essential and 2 optional items.^15^

The following steps were performed sequentially: 1) formulation of the guiding question and research objective; 2) development of the search strategy; 3) literature search; 4) establishment of inclusion and exclusion criteria; 5) screening of articles by title and abstract reading; 6) screening oof articles by full text; 7) summary of results; and 8) discussion.

### Guiding question

The Population, Concept, and Context strategy was used to formulate the research question and was defined as follows: P: People with T1DM; C: Prevalence of autoimmune diseases; and C: Worldwide literature.

Based on these definitions, the following research question was formulated: “What is the prevalence of autoimmune diseases in people with type 1 diabetes mellitus as described in the global literature?”

### Search strategy

The search strategy was developed in collaboration with a librarian. While the protocol for this scoping review was not registered before initiating the process, all steps were conducted based on recognized methodological guidelines such as PRISMA-ScR.

We selected keywords and health descriptors (DeCS/MeSH) related to each element of the research question: type 1 *diabetes mellitus*, epidemiology, autoimmune diseases, and prevalence. The Boolean operator AND was used to combine the four main elements of the search strategy, while the Boolean operator OR was used to include variations of terms related to each element, such as “autoimmune disease” OR “autoimmune diseases.”

### Literature search

The search strategy was applied to the following databases: PubMed, Embase, Scopus, Lilacs, and Web of Science. The final search was conducted across all databases on July 12, 2023.

A bibliographic survey was conducted in July 2023. The detailed search strategy used in the databases is as follows:

(‘prevalence’/exp OR prevalence) AND (‘autoimmune diseases’/exp OR ‘autoimmune diseases’ OR ‘autoimmune disease’/exp OR ‘autoimmune disease’) AND (‘diabetes mellitus, type 1’/exp OR ‘diabetes mellitus, type 1’ OR ((‘diabetes’/exp OR diabetes) AND mellitus AND type AND (‘1’/exp OR 1))) AND (‘epidemiology’/exp OR epidemiology)

No searches were conducted in other sources, such as reports.

A total of 6,793 articles were identified across the following databases: PubMed (n=2,510), Embase (n=2,528), Scopus (n=1,423), Lilacs (n=264), and Web of Science (n=68). We then applied database search filters to select articles published from 2018 onwards. This timeframe was selected because of the existence of a previous scoping review with a similar guiding question, published in 2018.^16^ After applying this exclusion criterion, 2,146 articles remained: PubMed (n=656), Embase (n=1,010), Scopus (n=399), Lilacs (n=51), and Web of Science (n=30).

The results were downloaded in CSV format, and duplicates were removed using Deduplicator, leaving 1,770 articles. Subsequently, some articles were excluded for reasons such as lack of full text, conference abstracts instead of published articles, or duplicates that were not automatically removed by the software. After exclusions, 1,751 articles remained for screening. The final dataset was transferred to a shared Google Sheets spreadsheet, where the authors collaboratively conducted the screening process.

### Eligibility criteria

The included studies were original research articles and guidelines, published in English, Spanish, and Portuguese that addressed the research question and were issued between 2018 and July 2023. These languages were selected because they are spoken and understood by all the authors, ensuring that the research team could adequately review, interpret, and analyze the studies.

Studies were excluded if they did not present data on the prevalence of autoimmune diseases in patients with T1DM, were published in languages other than those selected, or were published outside the proposed timeframe. We also excluded studies that did not have a summary or full article available in the databases, as well as those that did not report the prevalence of autoimmune diseases, sample selection criteria, or diagnostic criteria.

### Selection of studies

The screening process was conducted by four authors who independently reviewed the titles and abstracts of 1,751 articles. Each article was categorized as “yes,” “no,” or “maybe” based on its relevance to the study’s inclusion criteria. Full-text copies of the articles classified as “yes” and “maybe” were obtained for further assessment.

At least two authors evaluated each full-text article to determine whether it met the inclusion criteria. Any disagreements were resolved by discussion and consensus. When necessary, a third author was consulted to reach the final decision.

**Figure 1:**
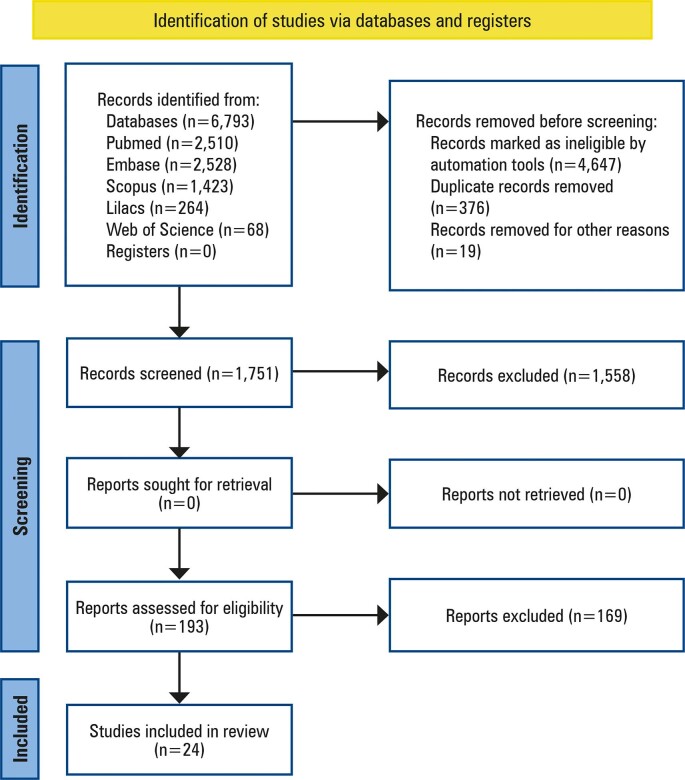
Flowchart of the study identification and selection process

At the end of this process, 24 articles met the pre-established inclusion criteria and were included in this study. Figure 1 shows a flowchart of the search process. The 24 selected studies were labeled as articles and numbered from 1 to 24.

### Data charting

Data were extracted using a standardized form developed by the authors on Google Sheets, which included the following information: article title, year of publication, country of origin, journal, study type, sample size (n), sample age range, and autoimmune diseases assessed. The articles were then classified according to the type of autoimmune disease associated with T1DM to facilitate the discussion of the results related to each disease. This classification is used in the discussion section. Following the classification, additional data were extracted, including disease prevalence, sex-based predominance, diagnostic criteria, and other relevant observations. Data extraction was performed in duplicate to ensure accuracy. Any discrepancies were resolved through discussion and consensus among the authors, and, when necessary, a third author was consulted.

### Results discussion and synthesis

The results were analyzed and presented in tables and narrative reports to ensure objective data extraction.

No formal critical appraisal of individual sources of evidence was conducted in this scoping review. Since the primary aim was to map the prevalence of autoimmune diseases in individuals with T1DM, the focus was on descriptive analysis and synthesis of the data rather than on assessing the methodological quality of each study. Studies were selected based on their relevance to the research question and the availability of data on the prevalence of autoimmune diseases, sample selection criteria, and diagnostic criteria.

## RESULTS

### Selection of sources of evidence

Twenty-four articles that met the inclusion criteria were selected. Most of the studies, including cross-sectional studies (n=16), retrospective studies (n=7), and prospective cohort studies (n=1), presented their results using descriptive statistics and varied sample sizes. The studies were published between 2018 and 2023 and were mainly conducted in single centers in Europe (n=6), Asia (n=11), Africa (n=1), Oceania (n=1), South America (n=2), and North America (n=1). Additionally, two multicenter studies were included: one was based on a review of medical records conducted in the United States, and the other was carried out across multiple health centers in Europe, North America, Asia, and Oceania.^26^

### Characteristics of sources of evidence

Most study results were presented using descriptive statistics and varied sample sizes and age characteristics of the evaluated patients (children, adolescents, and adults with T1DM). Table 1 shows the studies according to the authorship, associated autoimmune diseases, year of publication, journal, country of publication, study design, population, and study sample.

**Table 1:** Studies categorized according to authorship, associated autoimmune diseases, year of publication, journal, country of publication, study design, population, and study sample

Author and Year	Associated autoimmune diseases	Year	Journal	Country	Study design	Population and sample
Wang et al. 2023^(^^17^^)^	Autoimmune thyroiditis	2023/Jan.	Frontiers in Endocrinology	Taiwan	Retrospective cohort study	761 children and adolescents with T1DM
Gimenez- Perez et al. 2022^(^^14^^)^	Autoimmune thyroiditis Celiac disease Rheumatoid arthritis Primary adrenal insufficiency Gastrointestinal diseases: Autoimmune atrophic gastritis or pernicious anemia and Ulcerative colitis Hepatobiliary diseases: Primary biliary cirrhosis	2022/Sept.	Diabetes Research and Clinical Practice	Spain	Cross-sectional study	13571 adults with T1DM
Burbaud et al. 2022^(^^18^^)^	Autoimmune thyroiditis Celiac disease	2022/Jul.	Archives de Pédiatrie	France	Retrospective cohort study	179 children and adolescents with T1DM
Sharma et al. 2022^(^^19^^)^	Celiac disease	2022/Feb.	Pediatric Diabetes	India	Retrospective cohort study	398 children and adolescents with T1DM
Haris et al. 2021^(^^20^^)^	Celiac disease	2021/Aug.	Journal of Pediatric Endocrinology and Metabolism	Qatar	Cross-sectional study	1325 children and adolescents with T1DM
Khan et al. 2021^(^^21^^)^	Autoimmune thyroiditis	2021/Jul.	Journal of the Pakistan Medical Association	Pakistan	Cross-sectional study	161 children and adolescents with T1DM
Szcześniak et al. 2019^(^^22^^)^	Autoimmune thyroiditis	2019/Nov.	Archives of Medical Science	Ireland	Cross-sectional study	188 children and adolescents with T1DM
Aljulifi et al. 2021^(^^23^^)^	Celiac disease	2021/Apr.	Annals of Saudi Medicine	Saudi Arabia	Cross-sectional study	539 adolescents and adults with T1DM
Unal et al. 2021^(^^24^^)^	Celiac disease	2021/Feb.	Journal of Clinical Research in Pediatric Endocrinology	Türkiye	Retrospective cohort study	668 children and adolescents with T1DM
Wędrychowicz et al. 2021^(^^25^^)^	Celiac disease	2021/Feb.	Pediatric Endocrinology Diabetes and Metabolism	Poland	Cross-sectional study	880 children and adolescents with T1DM
Taczaniwska et al. 2021^(^^26^^)^	Celiac disease	2021/Jun.	Journal of Diabetes	North Europe, South Europe, North America/Canada, Australia/New Zealand, and Asia/East Middle	Cross-sectional study	57375 children and adolescents with T1DM
Agarwal et al. 2020^(^^27^^)^	Celiac disease	2020/Aug.	Indian Pediatrics	India	Retrospective cohort study	208 children and adolescents with T1DM
Głowińska-Olszewska et al. 2020^(^^28^^)^	Autoimmune thyroiditis Celiac disease	2020/Aug.	Frontiers in Endocrinology	Poland	Retrospective cohort study	493 children and adolescents with T1DM
Peters et al. 2020^(^^29^^)^	Autoimmune thyroiditis	2020/Jan.	Clinical Endocrinology	Australia	Prospective cohort study	130 adults with T1DM
Calcaterra et al. 2019^(^^30^^)^	Autoimmune thyroiditis	2019/Sept.	Hormone Research in Paediatrics	Italy	Cross-sectional study	166 children and adolescents with T1DM
Cardínez et al. 2019^(^^31^^)^	Autoimmune thyroiditis Celiac disease	2019/Jun.	Diabetes	Canada	Cross-sectional study	374 adults with T1DM
Puñales et al. 2019^(^^32^^)^	Celiac disease	2019/Jun.	Pediatric diabetes	Brazil	Cross-sectional study	881 children and adolescents with T1DM
Sharma et al. 2019^(^^3^^)^	Autoimmune thyroiditis Celiac disease Primary adrenal insufficiency	2019/Jan.	Indian Journal of Endocrinology and Metabolism	India	Cross-sectional study	150 children and adolescents with T1DM
Rinawi et al. 2019^(^^33^^)^	Celiac disease	2019/Jan.	Acta Paediatrica	Israel	Retrospective cohort study	425 children and adolescents with T1DM
Paruk et al. 2019^(^^34^^)^	Celiac disease	2019/Jan.	Journal of Gastroenterology and Hepatology	South Africa	Cross-sectional study	202 adolescents and adults with T1DM
Velasco- Benítez et al. 2018^(^^35^^)^	Celiac disease	2018/Dec.	Colomb Med (Cali)	Colombia	Cross-sectional study	155 children and adolescents with T1DM
Slae et al. 2019^(^^36^^)^	Celiac disease	2019/Feb.	Digestive Diseases and Sciences	Israel	Cross-sectional study	314 children, adolescents, and young adults with T1DM
Bao et al. 2019^(^^37^^)^	Autoimmune thyroiditis Celiac disease Rheumatoid arthritis Primary adrenal insufficiency Gastrointestinal diseases: Ulcerative colitis Crohn’s disease Hepatobiliary diseases: Primary biliary cirrhosis Primary sclerosing cholangitis Autoimmune hepatitis	2019/Apr.	Journal of Diabetes	USA	Cross-sectional study	158,865 adults with T1DM
Hwang et al. 2018^(^^12^^)^	Autoimmune thyroiditis	2018/Mar.	Annals of Pediatric Endocrinology and Metabolism	South Korea	Cross-sectional study	102 children and adolescents with T1DM

### Results of individual sources of evidence and synthesis of results

The included articles evaluated the association between T1DM and various autoimmune diseases, with the most commonly cited being autoimmune thyroiditis, celiac disease, rheumatoid arthritis, and primary adrenal insufficiency. Table 2 shows the autoimmune diseases identified, the number of articles that mentioned them, and the minimum and maximum prevalence.

**Table 2:** Most frequent autoimmune diseases associated with T1DM found in the selected articles

Autoimmune diseases	References	Fr (%)	Minimum prevalence %	Higher prevalence %
Autoimmune thyroiditis	17, 18, 21, 30-31, 3	6 (25)	5.50	41.20
Celiac disease	14-15, 17–20, 23–28, 31–37	19 (76)	0.45	24.80
Rheumatoid arthritis	14, 37	2 (8)	0.40	5.10
Primary adrenal insufficiency	14, 3, 37	3 (12)	0.60	2.60
Gastrointestinal diseases*	14, 37	2 (8)	1.07	3.71
Autoimmune atrophic gastritis or pernicious anemia	14	1 (4)	0.97	0.97
Ulcerative colitis	14, 37	2 (8)	0.34	0.99
Crohn’s disease	37	1 (4)	0.43	0.50
Hepatobiliary diseases	14, 37	2 (8)	0.13	0.58
Primary biliary cirrhosis	14, 37	2 (8)	0.09	0.25
Primary sclerosing cholangitis	37	1 (4)	0.17	0.28
Autoimmune hepatitis	37	1 (4)	0.11	0.17

*Except celiac disease.

Fr: Frequency of articles on the topic.

## DISCUSSION

### Autoimmune thyroiditis

Autoimmune thyroiditis includes a range of disorders, such as Hashimoto’s thyroiditis, which causes hypothyroidism, and Graves’ disease, which causes hyperthyroidism.

The pathophysiology of Hashimoto’s thyroiditis involves the destruction of thyroid follicular cells through the action of autoantibodies, such as anti-thyroid peroxidase (anti-TPO) and anti-thyroglobulin antibodies (anti-TG), which play a key role in confirming the diagnosis of thyroid autoimmunity. This chronic inflammatory process leads to progressive glandular fibrosis and impaired thyroid function, resulting in a gradual loss of functional thyroid cells and, consequently, a reduction in thyroid hormone production, culminating in hypothyroidism.^38^

In contrast, Graves’ disease is characterized by the presence of thyrotropin receptor autoantibodies (TSH-R-Abs), particularly the stimulatory subtype (TSAb), which activates the TSH receptor in a manner that mimics natural hormones, resulting in excessive thyroid hormone production.^39^

Autoimmune thyroid disease is the most prevalent autoimmune disease in individuals with T1DM,^26^ justifying annual screening for thyroid diseases in these individuals. This correlation may be partly explained by the shared predisposing genetic factors between the two conditions, particularly the HLA-DR3 DQ2 and HLA-DR4 DQ8 loci, which are part of the class II histocompatibility system.^39-41^ Genetic susceptibility related to other genes, such as the cytotoxic antigen associated with T lymphocytes (CTLA4), the non-receptor protein tyrosine phosphatase type 22 (PTPN22), the interleukin-2 receptor (IL2Ra), among others, has also been documented in other studies.^39-41^ HLA-DR7 has been identified as a protective genetic factor for Graves’ disease and Hashimoto’s thyroiditis,^40,42^ while the HLA-DR15 and HLA-D14 loci are associated with T1DM.^40^

The definitive diagnosis of hypothyroidism and hyperthyroidism is made by evaluating thyroid hormone levels. A reduction in free T4 levels and an increase in TSH levels are characteristic of hypothyroidism, whereas the opposite is observed in hyperthyroidism.^21^

In this review, 18 studies addressed the prevalence of autoimmune thyroid diseases in individuals with T1DM, reporting a prevalence range of 5.5% to 41.2%.^17,30,31,43-45^

Hashimoto’s disease was also highlighted in this review as the most commonly associated thyroid condition in individuals with T1DM, as noted by Burbaud et al.^18^ and Cardínez et al.^31^ In the first study, 15.6% of the children diagnosed with T1DM tested positive for at least one specific antibody against Hashimoto’s disease. In the second study, 40.5% of individuals with T1DM were also diagnosed with Hashimoto’s disease.

In contrast, Graves’ disease prevalence was not as high as that of Hashimoto’s disease (40.5% *versus* 2.7%).^31^ A similar low prevalence of 0.62% was reported in the study conducted by Khan et al.^21^

The most affected sex in all studies was female. Age also proved to be a determining factor: the older the patient, the greater the positivity for antithyroid antibodies and the greater the prevalence of thyroid diseases.^21^

### Celiac disease

Celiac disease is an autoimmune disorder associated with gluten intolerance. It occurs in genetically susceptible individuals, causing inflammation and atrophy of the intestinal mucosa and subsequently poor nutrient absorption. Celiac disease was the second most cited autoimmune disorder in the selected studies (n=16), with nine cross-sectional studies, seven retrospective cohort studies, and one prospective cohort study. These studies were conducted in Africa (n=1),^34^ North America (n=2),^31,37^ South America (n=2),^32,35^ Middle East (n=4),^20,23,33,36^ India (n=3),^3,19,27^ Europe (n=5),^14,18,25,24,28^ and one was a multicenter study that evaluated patients from several continents.^28^

There has been ongoing debate about the potential benefits and risks of screening for celiac disease in asymptomatic individuals, including those in high-risk groups, such as patients with T1DM. A report published by the United States Preventive Services Task Force found no evidence supporting the benefits of this screening.^46^ However, many specialized medical societies currently recommend screening for celiac disease in children with T1DM.^47-49^

Generally, the diagnostic and screening approaches in children involve serological screening to measure IgA antibodies against tissue transglutaminase (anti-TTG IgA). To minimize the risk of false negatives, the test should be performed in patients who have not yet started a gluten-free diet, and total IgA should also be measured to screen for possible IgA deficiency. If needed, a duodenal biopsy via upper digestive endoscopy is performed based on the serum levels of anti-TTG IgA. This procedure is generally recommended for patients with significantly elevated levels (three times above the upper normal limit) or mildly elevated levels (lower than three times the upper normal limit) who also present with suggestive symptoms (diarrhea, frequent abdominal pain, and poor weight gain). Additionally, serological tests for anti-endomysial antibodies might be recommended for patients who have slightly elevated levels of anti-TTG IgA and who show few or no symptoms.^50^ Furthermore, recent European guidelines suggest that very high levels of anti-TTG IgA (ten times above the upper normal limit) can be used as a diagnostic criterion without confirmation by duodenal biopsy.^25,47^

In this review, the prevalence of celiac disease in patients with T1DM varied significantly among studies, with the lowest being 0.45%^37^ and the highest being 24.8%.^3^ This variation can be explained by several factors, including age, diagnostic criteria, country/region where the study was conducted, and the study design. It is worth mentioning that only 6 of the 18 studies included adults in their sample.^14,23,31,34,36,37^ In these studies, the prevalence ranged from 0.45%^37^ to 5.73%.^36^ In contrast, studies that excluded adult patients reported a prevalence range from 2.58%^35^ to 24.8%.^3^

Regarding diagnostic criteria, most studies involved screening with anti-TTG IgA serology followed by confirmation with duodenal biopsy via endoscopic duodenal aspiration (EDA). Notably, some studies considered very high titers of anti-TTG IgA antibodies (in general, 10x the upper limit of normality) as a direct diagnosis of celiac disease, without the need for EDA with duodenal biopsy, as recommended by the European guidelines mentioned above.^18,25,27,36^ Other studies required diagnostic confirmation by biopsy, regardless of previous IgA anti-TTG antibody values. Furthermore, some studies disregarded total IgA deficiency as a confounding factor.^20,26-28,35-37^

There was a discrepancy concerning sex, with six studies indicating a higher prevalence in females;^3,14,25-27,31^ eight studies found no statistically significant association between the sexes.^23-25,27,28,32-34,36^ The remaining studies did not analyze sex differences.

### Autoimmune rheumatic diseases

Systemic autoimmune rheumatic diseases are connective tissue diseases that encompass various pathologies, including rheumatoid arthritis, psoriasis, systemic lupus erythematosus, and Sjögren’s syndrome.^51^ Of these diseases, rheumatoid arthritis is the most frequently associated with T1DM. It is typically identified through clinical evaluation and the measurement of rheumatoid factor and anti-citrullinated peptide antibodies.^2^ The prevalence of RA in patients with T1DM was addressed in five articles and ranged from 0.4%^14^ to 5.1%.^2^ It is noteworthy that female patients, older patients, and those with T1DM for a longer period had a higher risk of developing rheumatoid arthritis.^52,53^ These rheumatic diseases are chronic, generally mild, but often underdiagnosed and have harmful effects on patients’ quality of life when maintained for years without treatment.^30^ Therefore, evidence of an association between this condition and T1DM in clinical practice can facilitate its diagnosis and early initiation of treatment.

### Primary adrenal insufficiency

Primary adrenal insufficiency, or Addison’s disease, results from a rare autoimmune process involving the destruction of the adrenal cortex and is often associated with autoimmune polyglandular syndromes. Although more than half of patients with Addison’s disease have other autoimmune disorders, less than 2% of patients with autoimmune diseases have Addison’s disease.^54^ In our literature review, adrenal insufficiency was addressed in five studies that comprehensively evaluated autoimmune diseases. The diagnostic criteria included a reduced activity of the enzymes 21-hydroxylase and 17 alpha-hydroxylase;^2^ the requirement for glucocorticoids or mineralocorticoids therapy^31^ and the presence of a previous clinical-laboratory diagnosis or documentation in the medical record.^37^ All studies indicated a low prevalence of Addison’s disease in patients with T1DM, ranging from 0.6%^53^ to 2.6%.^2^ Notably, Addison’s disease is reported to be independent of sex and ethnicity.^14,37,53^ Despite its low incidence, Addison’s disease is a potentially lethal pathology, and screening is recommended immediately after the diagnosis of T1DM.^52^

### Gastrointestinal and hepatobiliary diseases

In addition to Celiac Disease, two studies evaluated the prevalence of atrophic gastritis/pernicious anemia, Crohn’s disease, and ulcerative colitis.^13,14^ The prevalences found were 0.34-0.58% for ulcerative colitis, 0.43% for Crohn’s disease, and 0.97% for autoimmune atrophic gastritis.

The same studies evaluated the prevalence of autoimmune disorders related to liver and bile ducts, reporting a prevalence of 0.09-0.10% for primary biliary cirrhosis, 0.28% for primary sclerosing cholangitis, and 0.11% for autoimmune hepatitis.

### Other autoimmune diseases

Five reviewed studies also associated T1DM with disorders affecting the skin, hair, musculoskeletal system, neurological system, or gonads. Similar to the patterns observed in autoimmune thyroiditis, an increased prevalence of these autoimmune disorders was noted in women and older patients.^14,37^

Skin diseases generally showed slight variation in prevalence, ranging from <1% to 3.8%, with psoriasis having the highest incidence, representing 2.7%, as Gimenez-Perez et al.^14^ pointed out.

Neurological disorders, such as multiple sclerosis, myasthenia gravis, uveitis, and autoimmune neuropathy, had a lower prevalence, with the highest reported prevalence of 0.5% reported by Cardínez et al.^31^in patients with T1DM and multiple sclerosis.

In contrast, the central gonadal disorder reported was ovarian insufficiency with a low prevalence and slight variation, ranging from 0% as reported by Gimenez-Perez et al. ^14^ to 0.6% as reported by Bao et al.^37^

Autoimmune hemolytic anemia is associated with a lower incidence. It was documented in one patient and corresponded to a prevalence of 0.7% in a study by Sharma et al.^3^

### Limitations

This scoping review has some limitations. First, the majority of the studies included in the analysis were cross-sectional. While these studies are valuable for estimating the prevalence of autoimmune diseases in patients with T1DM, they do not provide evidence to determine whether these conditions increase risk in the general population. However, as the primary objective of this review was to assess the prevalence of these diseases, those studies were appropriate for this purpose and established a basis for international comparisons.

Many of the selected articles relied on a methodology involving the review of medical records, with diagnoses made by physicians following local protocols instead of trained researchers. While these protocols were based on guidelines from international medical societies, their interpretation can vary among healthcare professionals, potentially introducing bias. This variability may result in inconsistencies in diagnoses and affect the accuracy of the results. Additionally, differences in the diagnostic protocols used could have influenced the observed prevalence rates.

Finally, the significant diversity of samples, including limited representation of adults and individuals from different countries and social classes, may introduce variability in the results and limit the generalizability of findings to other populations. This diversity of samples can influence the prevalence of the autoimmune diseases studied, as factors such as age, geographic location, and socioeconomic conditions can impact the incidence and severity of these diseases.

## CONCLUSION

This scoping review investigated the prevalence of autoimmune diseases in patients with T1DM. Among the selected articles, autoimmune thyroiditis-comprising Graves’ disease and Hashimoto’s thyroiditis-and celiac disease were the most frequently reported autoimmune diseases in patients with T1DM. Other conditions, such as rheumatoid arthritis, primary adrenal insufficiency, gastrointestinal disorders, and skin diseases, were reported less frequently.

The complexity of T1DM management increases in the presence of multiple autoimmune comorbidities, emphasizing the need for a comprehensive approach to care for these patients. Therefore, the results of this review highlight the importance of future research to elucidate the relationship between T1DM and various autoimmune pathologies. A deeper understanding of these associations is vital to optimize the clinical management and quality of life of these patients and guide the development of public health policies, screening strategies, and educational initiatives tailored to the specific needs of this population.
